# The role of small airway function parameters in preschool asthmatic children

**DOI:** 10.1186/s12890-023-02515-3

**Published:** 2023-06-20

**Authors:** Liangqin Yi, Yan Zhao, Ziyao Guo, Qinyuan Li, Guangli Zhang, Xiaoyin Tian, Ximing Xu, Zhengxiu Luo

**Affiliations:** 1 Chongqing Key Laboratory of Pediatrics, International Science and Technology Cooperation base of Child Development and Critical Disorders, Department of Children’s Hospital of Chongqing Medical, Key Laboratory of Child Development and Disorders, National Clinical Research Center for Child Health and Disorders, Department of Clinical Laboratory center, University of Education, 400014 Chongqing, China; 2 National Clinical Research Center for Child Health and Disorders, Department of Respiratory Medicine, Children’s Hospital of Chongqing Medical University, Chongqing, China; 3 Big Data Center for Children’s Medical Care, Children’s Hospital of Chongqing Medical University, Chongqing, China

**Keywords:** Asthma, Small airway function, Spirometry, FEF25-75%, FEF50%, FEF75%

## Abstract

**Background:**

Small airways are the major sites of inflammation and airway remodeling in all severities of asthma patients. However, whether small airway function parameters could reflect the airway dysfunction feature in preschool asthmatic children remain unclear. We aim to investigate the role of small airway function parameters in evaluating airway dysfunction, airflow limitation and airway hyperresponsiveness (AHR).

**Methods:**

Eight hundred and fifty-one preschool children diagnosed with asthma were enrolled retrospectively to investigate the characteristics of small airway function parameters. Curve estimation analysis was applied to clarify the correlation between small and large airway dysfunction. Spearman’s correlation and receiver-operating characteristic (ROC) curves were employed to evaluate the relationship between small airway dysfunction (SAD) and AHR.

**Results:**

The prevalence of SAD was 19.5% (166 of 851) in this cross-sectional cohort study. Small airway function parameters (FEF25-75%, FEF50%, FEF75%) showed strong correlations with FEV_1_% (r = 0.670, 0.658, 0.609, *p*<0.001, respectively), FEV_1_/FVC% (r = 0.812, 0.751, 0.871, *p*<0.001, respectively) and PEF% (r = 0.626, 0.635, 0.530, *p*<0.01, respectively). Moreover, small airway function parameters and large airway function parameters (FEV_1_%, FEV_1_/FVC%, PEF%) were curve-associated rather than linear-related (*p*<0.001). FEF25-75%, FEF50%, FEF75% and FEV_1_% demonstrated a positive correlation with PC_20_ (r = 0.282, 0.291, 0.251, 0.224, *p*<0.001, respectively). Interestingly, FEF25-75% and FEF50% exhibited a higher correlation coefficient with PC_20_ than FEV_1_% (0.282 vs. 0.224, *p* = 0.031 and 0.291 vs. 0.224, *p* = 0.014, respectively). ROC curve analysis for predicting moderate to severe AHR showed that the area under the curve (AUC) was 0.796, 0.783, 0.738, and 0.802 for FEF25-75%, FEF50%, FEF75%, and the combination of FEF25-75% and FEF75%, respectively. When Compared to children with normal lung function, patients with SAD were slightly older, more likely to have a family history of asthma and airflow obstruction with lower FEV_1_% and FEV_1_/FVC%, lower PEF% and more severe AHR with lower PC_20_ ( all *p*<0.05).

**Conclusion:**

Small airway dysfunction is highly correlated with large airway function impairment, severe airflow obstruction and AHR in preschool asthmatic children. Small airway function parameters should be utilized in the management of preschool asthma.

**Supplementary Information:**

The online version contains supplementary material available at 10.1186/s12890-023-02515-3.

## Introduction

Asthma is a common chronic airway inflammatory disease affecting the entire bronchial tree. Recurrent and excessive airway inflammation could result in persistent airway epithelial injury, abnormal repairment, and airway remodeling, which accelerates both small and large airway function exacerbation [1]. The main goal of current asthma treatment is to achieve symptom control, and its long-term management aims to maintain optimal lung function [2]. The Global Initiative for Asthma (GINA) has established specific recommendations for spirometry parameters including forced expiratory volume in 1 s (FEV_1_) and peak expiratory flow (PEF) [2], which serve as indicators of large airway function and are used in the assessment and management of asthma.

Small airways are defined as the 7th-8th generation airways with an internal diameter of less than 2 mm with no cartilage in their walls [3], have been identified as the main sites of type 2 inflammation and airway remodeling in both adults and children with asthma [4–6]. Small airway dysfunction (SAD) has been associated with worse asthma control [7], increased exacerbations [8, 9], airway inflammation and airway hyperresponsiveness (AHR) [10, 11], increased risk for asthma development [12, 13], and loss of lung function with aging in children [14].

However, large airway function parameters are insensitive to evaluating SAD in the early stage of asthma, especially in most asthmatic children with normal or nearly normal FEV_1_% (≥ 80%) [15–17]. Therefore, it is necessary to identify and apply other spirometry parameters for asthmatic children that can evaluate small airway function sensitively. Studies [18–21] have demonstrated that forced expiratory flow between 25% and 75% of forced vital capacity predicted (FEF25-75%), forced expiratory flow at 50% of forced vital capacity predicted (FEF50%) and forced expiratory flow at 75% of forced vital capacity predicted (FEF75%) can be used to evaluate small airway function as these parameters are less effort-dependent than FEV_1_, PEF, forced expiratory volume in 1 s/forced expiratory vital capacity ratio (FEV_1_/FVC). Although evidence in adults showed a good correlation between large and small airway function [21], the correlation in asthmatic children remains unclear and whether small airway function parameters could reflect airway dysfunction need to be clarified [22, 23]. This study aims to investigate the role of small airway function parameters in evaluating airway dysfunction, airflow limitation and AHR in preschool asthmatic children.

## Materials and methods

### Patients

The observational, retrospective cohort study was conducted at the Children’s Hospital of Chongqing Medical University, a tertiary teaching hospital in Chongqing, China. Preschool children diagnosed with suspected asthma between January 1, 2019 and December 31, 2020 were enrolled retrospectively by reviewing the electronic medical databases from the Department of Respiratory. The inclusion criteria were all of the following: (i) clinical asthma diagnosis for the first time according to GINA guidelines(2018) [2] by at least one pediatric pulmonologist, (ii) age 3–5 years; (iii) the completion of standard lung function test with technically acceptable flow-volume curves [24], (iv) without respiratory infections for 4 weeks before lung function test [25]. The exclusion criterion was any of the following: (i) acute and/or chronic diseases that could affect lung function tests (including bronchiectasis, pulmonary tuberculosis, interstitial lung disease, heart failure, severe psychiatric disorders, etc.); (ii) use of anti-asthma therapy (including inhaled corticosteroids, leukotriene receptor antagonist and long-acting beta-agonists) ≥ 4 weeks or other medications that affecting lung function test [26, 27]. (iii) with poor-quality spirometric data.

### Lung function test and definitions

Spirometry (Masterscreen Paediatric; PFT) was performed according to the guidelines of the American Thoracic Society(ATS) and or European Respiratory Society (ERS) [24] by trained technicians at the lung function laboratory. Short-acting beta-agonists should be stopped at least 4 h, while long-acting beta-agonists should be stopped at least 12 h and inhaled corticosteroids should be discontinued at least 24 h before the performance of spirometry or either a bronchial provocation test (using methacholine) or a bronchodilator test (using salbutamol). Whether a bronchial provocation test was conducted depended on the patients’ condition (including FEV_1_ ≥ 70% and without dyspnea). All lung function tests were repeated at least three times to ensure reproducibility, and the best FVC maneuver from the three attempts was chosen. The professional investigator reviewed the volume-time and flow-volume tracings and exclude poor measurements. Reference values of spirometry parameters for children have been adjusted for age, height and sex [28]. Pre-bronchial provocation or pre-bronchodilator spirometry parameters and the value of the provocative concentration of methacholine that caused a 20% fall in FEV_1_ (PC_20_) were recorded and included in this analysis.

Airflow obstruction was defined by FEV_1_% and FEV_1_/FVC% [25, 29]. Specifically, normal (FEV_1_%≥80% and accompanied with FEV_1_/FVC% ≥ 92%), mild (70% ≤ FEV_1_%<80% or FEV_1_ ≥ 80% but FEV_1_/FVC%<92%), moderate (60% ≤ FEV_1_%<70%), moderate to severe (50% ≤ FEV_1_%<60%), severe (35% ≤ FEV_1_%<50%) and extremely severe ( FEV_1_%<35%).

The degree of AHR was categorized into three levels based on the PC_20_ [30, 31], borderline AHR ( 4 g/L<PC_20_ ≤ 16 g/L), mild AHR (1 g/L<PC_20_ ≤ 4 g/L), and moderate to severe AHR (PC_20_ ≤ 1 g/L).

FEV_1_% was used to assess large airway function, and its lower limit value is 80%. FEF25-75%, FEF50%, and FEF75% were used to evaluate small airway function, and their lower limit value is 65%. According to previous studies [18, 21] especially in the Chinese characteristics [28], SAD was defined as any two of these three small airway function parameters being<65% accompanied by FEV_1_% ≥ 80% [32]. Normal lung function (NLF) was defined as FEV_1_%≥80% and at least two of FEF25-75%, FEF50% and FEF75% ≥ 65%. Large airway dysfunction (LAD) was defined as FEV_1_%<80% and at least two of FEF25-75%, FEF50% and FEF75% ≥ 65%. Both large and small airway dysfunction (LSAD) was defined as FEV_1_%<80% and at least two of FEF25-75%, FEF50% and FEF75%<65%.

If there was at least one positive response to the common aeroallergens (house dust mites, cotton, cat and dog fur and birch, etc.) and food allergens (peanuts, milk, egg, mango, and shrimp, etc.) by skin prick test, a child were considered atopic [21]. Eosinophilia was defined as a patient having a peripheral blood eosinophil count of ≥ 0.5 × 10^9^/L and eosinophils comprising ≥ 5% of leukocytes [33].

### Data collection

Patients’ data were collected from the medical records by two trained researchers independently, using a standard collection form. A third researcher assisted in the data extraction if any disagreement existed. Demographic characteristics (sex, age, weight, height and body mass index (BMI)), family history of asthma (maternal and paternal history), comorbidity (allergic rhinitis and eczema), peripheral eosinophil count and proportion, skin prick test results, and baseline values of FVC, FEV_1_, FEV_1_/FVC, PEF, FEF25 (forced expiratory flow at 25% of FVC), FEF50, FEF75, FEF25-75 and PC_20_ were collected. All spirometry parameters were expressed as a percentage of predicted values (%pred).

### Statistical analysis

The distribution of continuous variables was assessed by the Shapiro-Wilk test, and continuous variables were expressed as the median and interquartile range (IQR), while categorical variables were presented as numbers and percentages (%). Continuous variables were assessed by the non-parametric Mann-Whitney U test, and Categorical variables were compared using the chi-square test with Fisher exact test or Monte Carlo method. Bonferroni correction was applied when multiple means needed to be compared. The prevalence of FEF25-75%, FEF50%, and FEF75% between subgroups was compared using the related samples Cochran’s test and McNemar’s test. Spearman’s correlation and correlation coefficient comparison analysis were used to evaluate the variables’ relation. Additionally, Curve Estimation analysis was performed for large airway function parameters (FEV_1_%, FEV_1_/FVC%, PEF%) and small airway function parameters (FEF25-75%, FEF50%, FEF75%) in the overall group and subgroups, which included 11 models: Linear, Logarithmic, Inverse, Quadratic, Cubic, Compound, Power, S, Growth, Exponential, and Logistic model. The best-fitting model was used to present the correlation among variables. Receiver-operating characteristic (ROC) curves were constructed to estimate the ability of small airway parameters in predicting AHR.

All data analyses were performed using the IBM SPSS software for Windows, (version 26.0 SPSS Inc. Chicago, IL, USA). A *p*-value of < 0.05 (two-tailed) was considered statistically significant. All graphics were completed by GraphPad Prism (Version 9.0.0 San Diego, California, USA).

## Results

### Characteristics

**Fig. 1:**
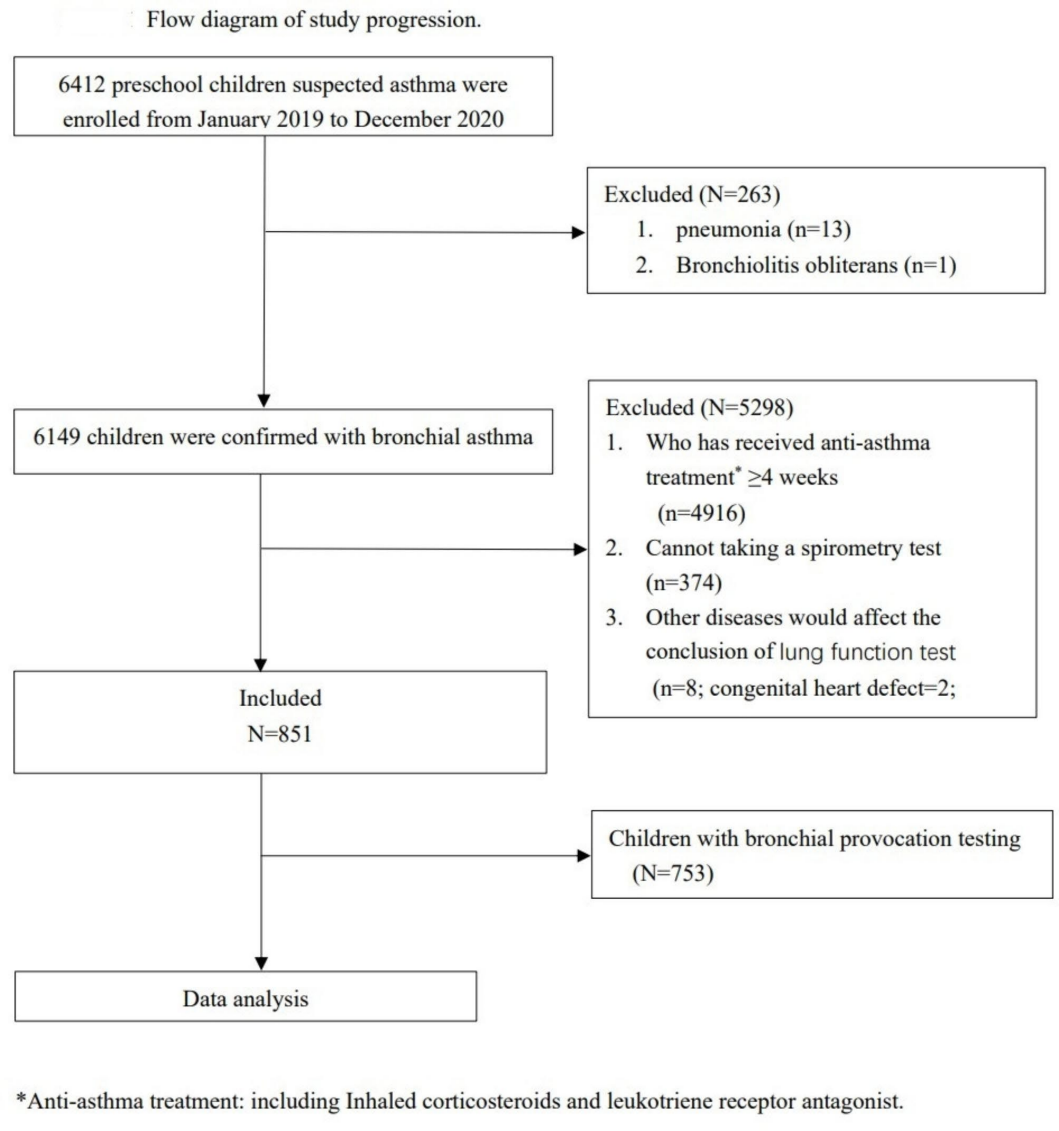
Flow diagram of study progression

The medical record database of the Department of Respiratory at the Children’s Hospital of Chongqing Medical University was screened. From January 1, 2019, to December 31, 2020, a total of 6412 preschool children with suspected asthma who presented with recurrent wheezing with or without chronic cough were identified and assessed in respiratory clinics. Out of these, 6149 children were diagnosed with bronchial asthma. Finally, 851 preschool asthmatic children who met the inclusion and exclusion criteria were enrolled in the study (Fig. 1).

In this study cohort, the median age of those 851 preschool asthmatics was 4.3 years old, and boy predominance of 57.5% (489 of 851). The majority of the children (91.5%, 779 of 851) had normal FEV1% (≥ 80%) and FEV1/FVC% (> 70%). The prevalence of abnormal small airway function parameters (FEF25-75%, FEF50%, FEF75%) was 25.4% (261 of 851), 27.4% (233 of 851), and 36.4% (310 of 851), respectively. The prevalence of SAD was 19.5% (166 of 851), the prevalence of NLF, LAD and LASD was 72.0% (613 of 851), 0.7% (6 of 851) and 7.8% (66 of 851), respectively. FEF75% showed the highest abnormal rate compared to FEF25-75% and FEF50% among the overall, NLF and SAD groups in preschool asthmatic children (*p*<0.001, as shown in Additional file 1: Appendix Fig. 1). Most of the patients had normal pulmonary ventilation (86.0%, 732/851), with mild, moderate, moderate to severe, severe and extremely severe airflow obstruction accounting for 10.0% (85/851), 1.9% (16/851), 1.6% (14/851), 0.4% (3/851), 0.1% (1/851), respectively. Among the 753 preschool children who performed bronchial provocation tests, 40.1% (302), 52.7% (397), and 7.2% [54] had borderline, mild, and moderate to severe AHR, respectively. More demographic, history and lung function information for this cohort was presented in Table 1.

**Table 1:** Demographic and lung function parameters in preschool asthmatic children (N = 851)

Characteristics	Median (P25, P75) or Number (%)
Age of asthma onset (y)	4.3 (3.9, 5.0)
Sex (boy)	489 (57.5)
BMI	16.0 (15.2, 17.0)
Family and personal history	
Parental wheeze	82 (9.6)
allergic rhinitis	412 (48.4)
eczema	226 (26.6)
atopy	489 (57.5)
Blood eosinophil test	483 (56.8)
Eosinophilia	190 (39.3)
Spirometry	
Patients with FEV_1_%≥80%	779 (91.5)
Patients with FEF25-75%<65%	216 (25.4)
Patients with FEF75%<65%	310 (36.4)
Patients with FEF50%<65%	233 (27.4)
Normal lung function (NLF)	613 (72.0)
Large airway dysfunction (LAD)	6 (0.7)
Small airway dysfunction (SAD)	166 (19.5)
Large and small airway dysfunction (LSAD)	66 (7.8)
FVC%	94.3 (86.7,103.7)
FEV_1_%	98.9 (90.2,108.0)
FEV_1_/FVC%	104.9 (99.0,110.7)
PEF%	85.4 (76.2,96.3)
FEF25%	84.5 (73.7,96.4)
FEF50%	80.0 (63.5,96.8)
FEF75%	73.4 (55.0,96.6)
FEF25-75%	81.0 (64.5,99.3)
Airflow Obstruction	
normal	732 (86.0)
mild	85 (10.0)
moderate	16 (1.9)
moderate to severe	14 (1.6)
severe	3 (0.4)
extremely severe	1 (0.1)
Patients with bronchial provocation testing	753 (88.5)
PC_20_ (g/L)	2.0 (2.0,8.0)
Airway Hyperresponsiveness (AHR) Borderline Mild Moderate to severe	753 (88.5) 302 (40.1) 397 (52.7) 54 (7.2)

BMI, Body mass index; FVC%, forced expiratory vital capacity in predicted; FEV_1_%, forced expiratory volume in 1 s in predicted; FEV_1_/ FVC%, forced expiratory vital capacity/forced expiratory vital capacity ratio; PEF%, peak expiratory flow in predicted; FEF25%, forced expiratory flow at 25% of FVC predicted; FEF50%, forced expiratory flow at 50% of FVC predicted; FEF75%, forced expiratory flow at 75% of FVC predicted; FEF25-75%, forced expiratory flow between 25% and 75% of FVC predicted; PC_20_, value of the provocative concentration of methacholine that causing a 20% fall in FEV_1_; AHR: Airway hyperresponsiveness.

**Fig. 2:**
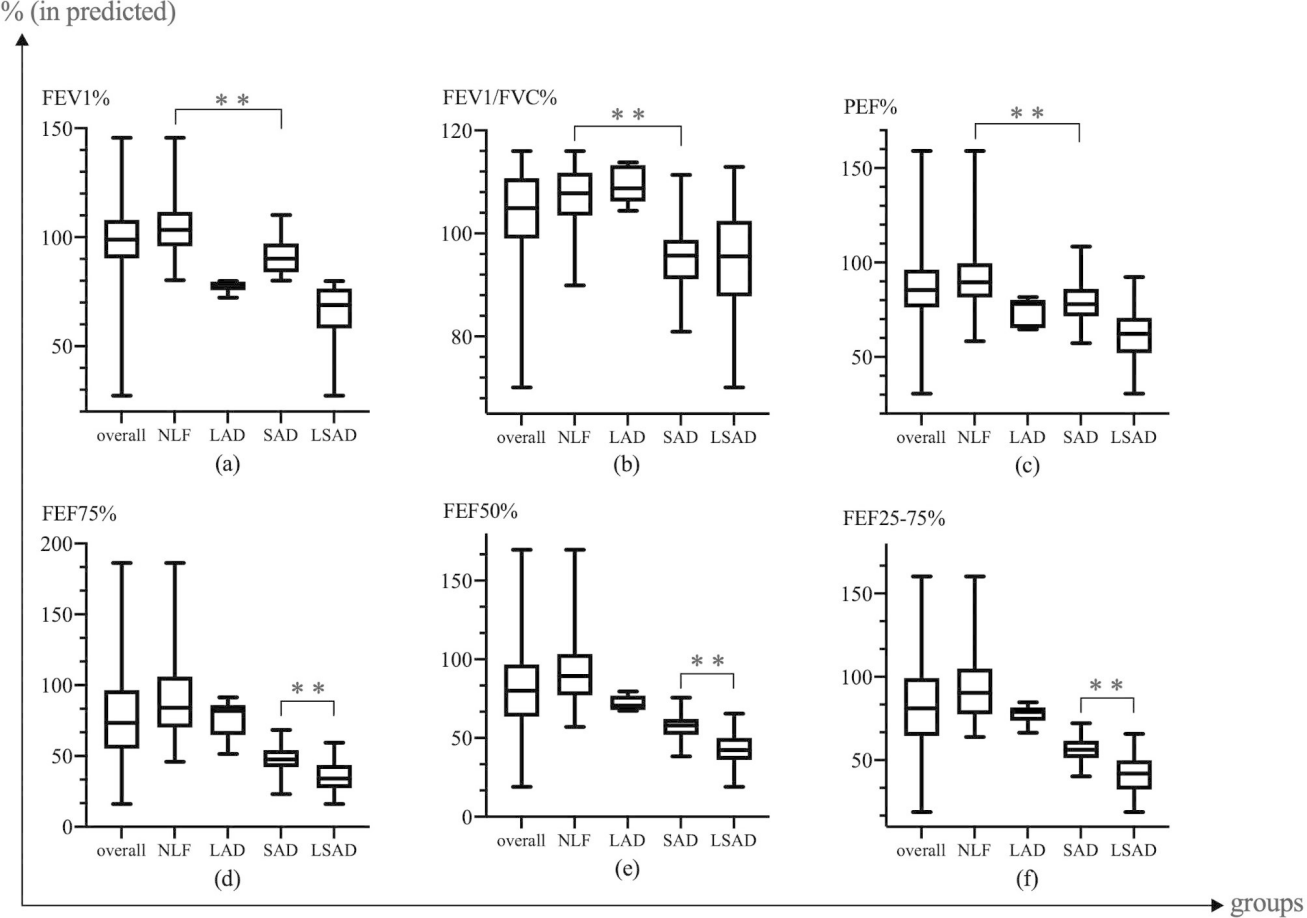
Comparison of spirometry parameters values among overall and subgroups. ─ Represent sprometric parameter, Spearman correlation coefficient, p; a1, The relationships among FEF25-75%, FEF50%, FEF75% and FEV1% in overall group; a2, The relationships among FEF25-75%, FEF50%, FEF75% and FEV1/FVC% in overall group; a3, The relationships among FEF25-75%, FEF50%, FEF75% and PEF% in overall group; b1, The relationships among FEF25-75%, FEF50%, FEF75% and FEV1% in NLF group; b2, The relationships among FEF25-75%, FEF50%, FEF75% and FEV1/FVC% in NLF group; b3, The relationships among FEF25-75%, FEF50%, FEF75% and PEF% in NLF group; c1, The relationships among FEF25-75%, FEF50%, FEF75% and FEV1% in LAD group; c2, The relationships among FEF25-75%, FEF50%, FEF75% and FEV1/FVC% in LAD group; c3, The relationships among FEF25-75%, FEF50%, FEF75% and PEF% in LAD group; d1, The relationships among FEF25-75%, FEF50%, FEF75% and FEV1% in SAD group; d2, The relationships among FEF25-75%, FEF50%, FEF75% and FEV1/FVC% in SAD group; d3, The relationships among FEF25-75%, FEF50%, FEF75% and PEF% in SAD group; e1, The relationships among FEF25-75%, FEF50%, FEF75% and FEV1% in LSAD group; e2, The relationships among FEF25-75%, FEF50%, FEF75% and FEV1/FVC% in LSAD group; e3, The relationships among FEF25-75%, FEF50%, FEF75% and PEF% in LSAD group; EV1%, forced expiratory volume in 1 s in predicted; FEV1/ FVC%, FEV1/ FVC%, forced expiratory vital capacity/forced expiratory vital capacity ratio; PEF%, peak expiratory flow in predicted; FEF50%, forced expiratory flow at 50% of FVC predicted; FEF75%, forced expiratory flow at 75% of FVC predicted; FEF25-75%, forced expiratory flow between 25% and 75% of FVC predicted; NLF, normal lung function; LAD, large airway dysfunction; SAD; small airway dysfunction; LSAD, large and small airway dysfunction

In the subgroups, asthmatic children in the SAD group were slightly elder, more likely to have a family asthmatic history, and severe airflow obstruction, as well as decreased PEF% and a higher degree of AHR with lower PC_20_ value as compared to NLF group. A similar tendency was observed in the comparison of the NLF and LSAD groups (*p*<0.005, Table 2). Furthermore, preschool patients in the NLF and SAD groups had normal FEV_1_% (≥ 80%), but patients in the SAD group had significantly lower FEV_1_% than those in the NLF group, as well as lower FEV_1_/FVC%, PEF% (*p*<0.001, Fig. 2a, b, c). Interestingly, patients with LSAD had the lowest FEF25-75%, FEF50%, and FEF75% among subgroups ((*p*<0.001,Fig. 2d, e, f). However, there were no differences among subgroups in terms of gender, history of allergic rhinitis, eczema, eosinophilia and atopic status (*p*>0.05).

**Table 2:** Clinical and lung function characteristics of asthmatic subjects in overall and subgroups

Characteristics	Overall N = 851 (100.0)	NLF N = 613 (72.0)	LAD N = 6 (0.7)	SAD N = 166 (19.5)	LSAD N = 66 (7.8)	P value (Subgroups)	P value (NLF vs. SAD)	P value (NLF vs. LSAD)
Age of asthma onset (y)	4.3 (3.9,5.0)	4.3 (3.8,4.9)	4.5 (3.8,5.0)	4.5 (4.0,5.3)	4.6 (4.1,5.1)	<0.001	0.002	0.024
Sex (boy)	489 (57.5)	347 (56.6)	5 (83.3)	100 (60.2)	37 (56.1)	0.519^**†**^	0.401	0.932
BMI	16.0 (15.2,17.0)	16.1 (15.2,17.1)	15.7 (14.5,16.8)	16.0 (15.2,17.0)	15.5 (14.6,16.4)	0.026	1.000	0.020
Family and Personal History								
Parental wheeze	82 (9.6)	51 (8.3)	0 (0.0)	25 (15.1)	6 (9.1)	0.076^**†**^	0.009	0.830
Allergic rhinitis	412 (48.4)	295 (48.1)	4 (66.7)	84 (50.6)	29 (43.9)	0.647^**†**^	0.571	0.518
Eczema	226 (26.6)	165 (26.9)	3 (50.0)	39 (23.5)	19 (28.8)	0.402^**†**^	0.374	0.745
Atopy	489 (57.5)	360 (76.3)	2 (66.7)	91 (70.0)	36 (76.6)	0.437^**†**^	0.144	0.960
Eosinophilia	190 (39.3)	137 (39.5)	2 (40.0)	33 (37.1)	18 (42.9)	0.936^**†**^	0.678	0.739
Parameters of spirometry								
FVC%	94.3 (86.7,103.7)	96.0 (88.8,105.4)	69.0 (68.5,73.4)	94.8 (88.1,102.0)	75.0 (61.8,81.8)	<0.001	1.000	<0.001
FEV_1_%	98.9 (90.2,108.0)	103.4 (95.8,111.8)	78.4 (75.6, 79.7)	90.1 (83.9,97.2)	69.0 (58.1,76.5)	<0.001	<0.001	<0.001
FEV_1_/FVC%	104.9 (99.0,110.7)	107.8 (103.5,111.8)	108.8 (106.2,113.3)	95.7 (91.1,98.8)	95.6 (87.8,102.5)	<0.001	<0.001	<0.001
PEF%	85.4 (76.2,96.3)	89.5 (81.5,99.7)	78.0 (65.2,80.4)	77.9 (71.4,86.1)	62.2 (51.9,70.9)	<0.001	<0.001	<0.001
FEF25%	84.5 (73.7,96.4)	90.6 (81.5,100.9)	75.9 (66.8, 80.4)	71.2 (65.3,77.2)	54.5 (45.2,63.6)	<0.001	<0.001	<0.001
FEF50%	80.0 (63.5,96.8)	89.3 (77.1,103.4)	70.6 (67.7,77.0)	58.0 (51.9,62.1)	42.4 (36.1,50.2)	<0.001	<0.001	<0.001
FEF75%	73.4 (55.0,96.6)	84.1 (70.1,106.1)	81.8 (64.7,86.2)	47.6 (42.1,54.3)	34.1 (27.3,43.8)	<0.001	<0.001	<0.001
FEF25-75%	81.0 (64.5,99.3)	90.4 (77.5,105.1)	79.0 (73.6,81.7)	56.3 (51.2,61.9)	42.0 (32.3,50.0)	<0.001	<0.001	<0.001
Airflow obstruction						<0.001^**†**^	<0.001	<0.001
Normal	732 (86.0)	611 (99.7)	0	121 (72.9)	0			
Mild	85 (10.0)	2 (0.3)	6 (100.0)	45 (27.1)	32 (48.5)			
Moderate	16 (1.9)	0	0	0	16 (24.2)			
Moderate to severe	14 (1.6)	0	0	0	14 (21.2)			
Severe	3 (0.4)	0	0	0	3 (4.5)			
Extremely Severe	1 (0.1)	0	0	0	1 (1.5)			
PC_20_ (g/L)	2.0 (2.0,8.0)	2.0 (2.0,8.0)	8.0 (1.3,8.0)	2.0 (2.0,8.0)	2.0 (0.5,2.0)	<0.001	0.001	0.001
AHR^‡^						<0.001^**†**^	<0.001	<0.001
Borderline	302 (40.1)	248 (43.4)	3 (60.0)	46 (30.9)	5 (18.5)			
Mild	397 (52.7)	302 (52.8)	1 (20.0)	81 (54.4)	13 (48.1)			
Moderate to severe	54 (7.2)	22 (3.8)	1 (20.0)	22 (14.8)	9 (33.3)			

BMI, Body mass index; FVC%, forced expiratory vital capacity in predicted; FEV_1_%, forced expiratory volume in 1 s in predicted; FEV_1/_ FVC%, FEV_1_/FVC%, forced expiratory vital capacity/forced expiratory vital capacity ratio; PEF%, peak expiratory flow in predicted; FEF25%, forced expiratory flow at 25% of FVC predicted; FEF50%, forced expiratory flow at 50% of FVC predicted; FEF75%, forced expiratory flow at 75% of FVC predicted; FEF25-75%, forced expiratory flow between 25% and 75% of FVC predicted; PC_20_, value of the provocative concentration of methacholine that causing a 20% fall in FEV_1_; AHR: Airway hyperresponsiveness; Overall, the whole study population, NLF, normal lung function; LAD, large airway dysfunction; SAD; small airway dysfunction; LSAD, large and small airway dysfunction; P-value calculated using chi-square test (for categorical variables) or Mann-Whitney U test (for continuous variables); ^**†**^, data comparison of subgroups using Fisher exact test or Monte Carlo method (corrected by Bonferroni test); ^‡^, analysis in patients with bronchial provocation test.

### The correlation between FEF25-75%, FEF50%, FEF75% and FEV_1_%, FEV_1_/FVC%, PEF%

**Fig. 3:**
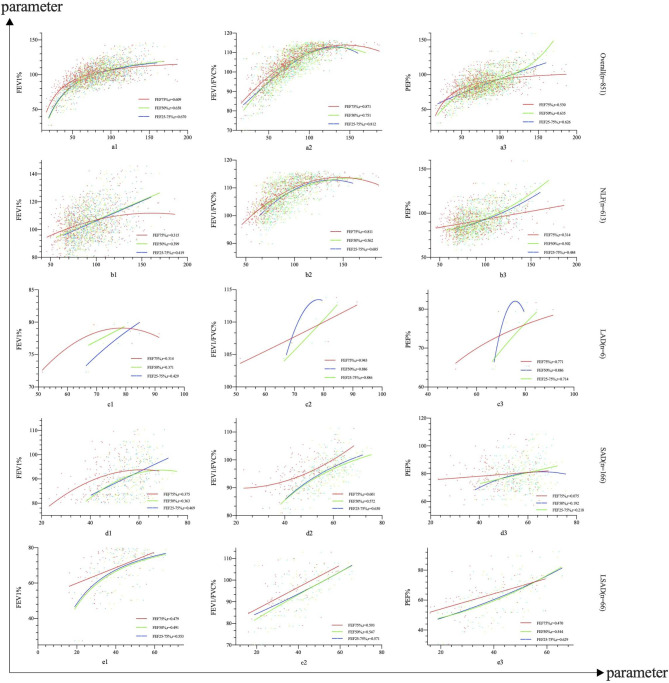
Relationships among small airway function parameters, FEV1%, FEV1/FVC% and PEF% of spirometry. ─ Represent sprometric parameter, Spearman correlation coefficient, p; a1, The relationships among FEF25-75%, FEF50%, FEF75% and FEV1% in overall group; a2, The relationships among FEF25-75%, FEF50%, FEF75% and FEV1/FVC% in overall group; a3, The relationships among FEF25-75%, FEF50%, FEF75% and PEF% in overall group; b1, The relationships among FEF25-75%, FEF50%, FEF75% and FEV1% in NLF group; b2, The relationships among FEF25-75%, FEF50%, FEF75% and FEV1/FVC% in NLF group; b3, The relationships among FEF25-75%, FEF50%, FEF75% and PEF% in NLF group; c1, The relationships among FEF25-75%, FEF50%, FEF75% and FEV1% in LAD group; c2, The relationships among FEF25-75%, FEF50%, FEF75% and FEV1/FVC% in LAD group; c3, The relationships among FEF25-75%, FEF50%, FEF75% and PEF% in LAD group; d1, The relationships among FEF25-75%, FEF50%, FEF75% and FEV1% in SAD group; d2, The relationships among FEF25-75%, FEF50%, FEF75% and FEV1/FVC% in SAD group; d3, The relationships among FEF25-75%, FEF50%, FEF75% and PEF% in SAD group; e1, The relationships among FEF25-75%, FEF50%, FEF75% and FEV1% in LSAD group; e2, The relationships among FEF25-75%, FEF50%, FEF75% and FEV1/FVC% in LSAD group; e3, The relationships among FEF25-75%, FEF50%, FEF75% and PEF% in LSAD group; EV1%, forced expiratory volume in 1 s in predicted; FEV1/ FVC%, FEV1/ FVC%, forced expiratory vital capacity/forced expiratory vital capacity ratio; PEF%, peak expiratory flow in predicted; FEF50%, forced expiratory flow at 50% of FVC predicted; FEF75%, forced expiratory flow at 75% of FVC predicted; FEF25-75%, forced expiratory flow between 25% and 75% of FVC predicted; NLF, normal lung function; LAD, large airway dysfunction; SAD; small airway dysfunction; LSAD, large and small airway dysfunction

Spearman’s correlation analysis revealed a relatively strong association between small and large airway function parameters. FEF25-75%, FEF50% and FEF75% were significantly correlated with FEV_1_% with a spearman coefficient of 0.670 (95%CI 0.629–0.710), 0.658 (95%CI 0.612–0.698) and 0.609 (95%CI 0.562–0.655), respectively (all *p*<0.001, see Fig. 3a1 and Additional File 2: Appendix Table 1). Similarly, FEF25-75%, FEF50% and FEF75% were correlated with FEV_1_/FVC% with a spearman coefficient of 0.812 (95%CI 0.786–0.838), 0.751 (95%CI 0.720–0.783) and 0.871 (95%CI 0.851–0.888), respectively (*p*<0.001, see Fig. 3a2 and Additional File 3: Appendix Table 2). Additionally, FEF25-75%, FEF50% and FEF75% were correlated with PEF% with a Spearman coefficient of 0.626 (95%CI 0.518–0.669), 0.635 (95%CI 0.589–0.676) and 0.530 (95%CI 0.481–0.579), respectively (*p*<0.01, see Fig. 3a3 and Additional File 4: Appendix Table 3). Furthermore, curve estimation analysis showed there was no linear relationship among FEF25-75%, FEF50% and FEF75% and FEV_1_%, FEV_1_/FVC% and PEF% (*p*<0.001, see Fig. 3a1-a3 and Additional File 5: Appendix Table 4 A to I for model comparisons).

Similar associations were observed in subgroups. Specifically, FEF25-75% showed a significant correlation with FEV_1_ in the NLF group (Spearman coefficient 0.419, 95%CI 0.344–0.485), the SAD group (Spearman coefficient 0.469, 95%CI 0.338–0.573), and the LSAD group (Spearman coefficient 0.553, 95%CI 0.364–0.693) (p<0.001). In the SAD group, FEF25-75% was significantly and robustly correlated with FEV_1_%, FEV_1_/FVC% and PEF% among small airway parameters (*p*<0.05). Similarly, FEF75% was significantly correlated with FEV_1_/FVC% with a Spearman coefficient of 0.811 (95%CI 0.780–0.838) in the NLF group, 0.601 (95%CI 0.495–0.701) in SAD group and 0.593 (95%CI 0.365–0.772) in LSAD group, respectively, (*p*<0.001). (All data were presented in Fig. 3b to e and Appendix Tables 1, 2 and 3). Moreover, the optimal models to show the correlation trends between variables in subgroups were presented in Fig. 3b to e (See Additional File 5: Appendix Table 4 A to I for detailed model comparisons).

### The correlation with PC_20_ and AHR in small airway function parameters

**Fig. 4:**
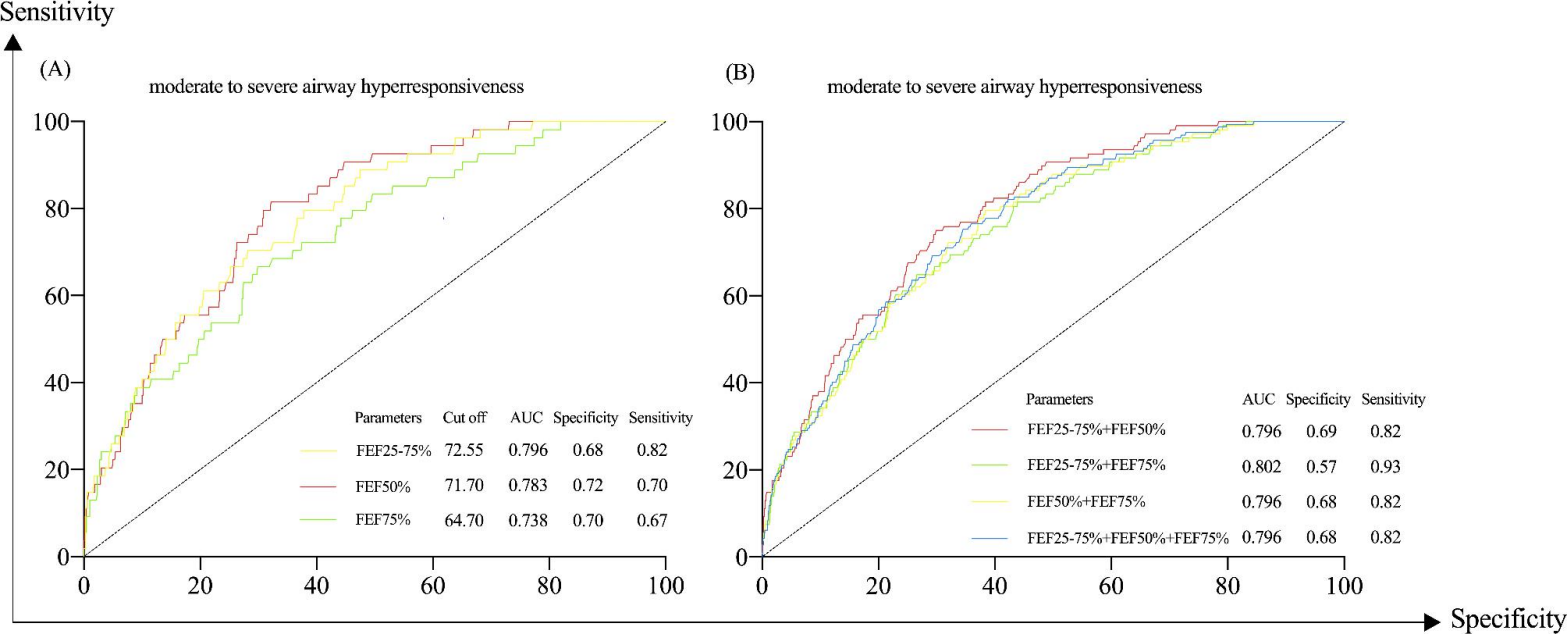
, ROC of small airway function parameters in predicting moderate to severe AHR (N = 753). **(A)** ROC of single small airway function parameter in predicting moderate to severe AHR; **(B)** ROC of combination of small airway function parameters in predicting moderate to severe AHR; ROC, receiver operating characteristic curve; FEF25-75%, forced expiratory flow between 25% and 75% of vital capacity; FEF50%, forced expiratory flow at 50% of vital capacity; FEF75%, forced expiratory flow at 75% of vital capacity; AHR, airway hyperresponsiveness

In 753 preschool patients who had performed bronchial provocation test, results demonstrated that FEV_1_%, FEF25-75%, FEF50%, and FEF75% were correlated with PC_20_ with the coefficient of 0.224 (95%CI 0.154–0.291), 0.282 (95%CI 0.206–0.350), 0.291 (95%CI 0.217–0.359), 0.251 (95%CI 0.174–0.321), respectively, (all *p*<0.001, Table 3), Of note, small airway function parameters, especially FEF50%, showed a slightly higher correlation level with PC_20_ than FEV_1_% (0.291 vs. 0.224, *p* = 0.014). The correlation patterns of PC_20_ with spirometry parameters varied among subgroups. In NLF group, weak positive correlations were observed between PC_20_ and FEV_1_% (r = 0.151, 95%CI 0.078–0.224), FEF25-75% (r = 0.249, 95%CI 0.165–0.322), FEF50% (r = 0.255, 95%CI 0.177–0.330) and FEF75% (r = 0.199, 95%CI 0.119–0.279) (all *p*<0.001). Interestingly, FEF50% and FEF25-75% showed higher coefficients with PC_20_ than FEV_1_% (0.255 vs. 0.151, *p* = 0.016, 0.249 vs. 0.151, *p* = 0.021). However, the correlation was absent for FEF75% with PC_20_ in the SAD group (r = 0.137, *p* = 0.096). Surprisingly, none of FEV_1_%, FEF50% and FEF25-75% had a significant relation with PC_20_ in LSAD group (*p*>0.05). Nevertheless, FEF75% still showed a moderate positive correlation with PC_20_ (r = 0.511, 95%CI 0.194–0.758, *p* = 0.006) in the LSAD group (all data were presented in Table 3). After adjustment for FEV_1_%, the correlations of PC_20_ with small airway parameters were slightly reduced but still existed (all *p*<0.05). Furthermore, ROC curves for FEF25-75%, FEF50% and FEF75% in predicting moderate to severe AHR showed areas under the curve (AUCs) was 0.796, 0.783, 0.738, respectively, with the optimal cut-off for FEF25-75% was 72.55% (specificity 0.68, sensitivity 0.82), for FEF50% was 71.7% (specificity 0.72, sensitivity 0.70), and for FEF75% was 64.7% (specificity 0.70, sensitivity 0.67). When combined, FEF25-75% and FEF75% had a higher AUC of 0.802 in predicting moderate to severe AHR, with a specificity of 0.57 and sensitivity of 0.93 (Fig. 4).

**Table 3:** The correlation with PC20 in spirometry parameters and the comparison of correlation coefficients

	Overall(n = 851) r (95%CI)	*p*	NLF(N = 613) r (95%CI)	*p*	LAD(N = 6) r	*p*	SAD(N = 166) r (95%CI)	*p*	LSAD(N = 66) r (95%CI)	*p*
FEV_1_%	0.224 (0.154–0.291)^**†**^	<0.001	0.151 (0.078–0.224)^**†**^	<0.001	-0.783	0.118	0.173 (0.016–0.335)^‡^	0.035	-0.020 (-0.471-0.402)	0.923
FEV_1_/FVC%	0.206 (0.129–0.278)	<0.001	0.143 (0.060–0.227)	0.001	-0.112	0.858	0.096 (-0.082-0.253)	0.245	0.367 (-0.046-0.702)	0.059
PEF%	0.179 (0.104–0.251)	<0.001	0.154 (0.078–0.231)	<0.001	-0.447	0.450	-0.025 (-0.191-0.151)	0.766	0.204 (-0.259-0.619)	0.308
FEF50%	0.291 (0.217–0.359)^‡^	<0.001	0.255 (0.177–0.330)^‡^	<0.001	0.112	0.858	0.264 (0.111–0.419)^‡^	0.001	0.171 (-0.254-0.543)	0.393
FEF75%	0.251 (0.174–0.321)^**†**^	<0.001	0.199 (0.119–0.279)^**†**^	<0.001	0.112	0.858	0.137 (-0.034-0.301)	0.096	0.511 (0.194–0.758)	0.006
FEF25-75%	0.282 (0.206–0.350)^‡^	<0.001	0.249 (0.165–0.322)^‡^	<0.001	0.112	0.858	0.207 (0.047–0.354)^‡^	0.011	0.332 (-0.021-0.621)	0.091

^**†**^, ^‡^: the different symbol in the same column indicated a significant difference between the two correlation coefficients (*p*<0.05), same letters indicated no difference between the two correlation coefficients (*p*>0.05); FEV_1_%, forced expiratory volume in 1 s in predicted; FEV_1_/ FVC, FEV1/ FVC%, forced expiratory vital capacity/forced expiratory vital capacity ratio; PEF, peak expiratory flow, FEF50%, forced expiratory flow at 50% of vital capacity; FEF75%, forced expiratory flow at 75% of vital capacity; FEF25-75%, forced expiratory flow between 25% and 75% of vital capacity; PC_20_, value of the provocative concentration of methacholine that causing a 20% fall in FEV_1_; Overall, the whole study population, NLF, normal lung function; LAD, large airway dysfunction; SAD; small airway dysfunction; LSAD, large and small airway dysfunction.

## Discussion

This study demonstrated that FEF75% was the most significantly decreased parameter of small airway function parameters measured by spirometry in these preschool asthmatic children, despite 91.5% (779 of 851) of these patients had FEV_1_% ≥ 80%. The prevalence of SAD was 19.5%, and the incidence of LSAD was 7.8%. Children with SAD were more likely to have a family history of asthma, more severe airflow obstruction, higher level of AHR and lower values of FEV_1_%, FEV_1_/FVC%, PEF% and PC_20_ compared to those with NLF. Further analysis showed that FEF25-75%, FEF50% and FEF75% were strongly correlated with FEV_1_%, FEV_1_/FVC% and PEF%. Additionally, Small airway function parameters were found to be correlated with PC_20_ and were good predictors for moderate to severe AHR. In general, the study suggested that small airway function parameters were associated with airway dysfunction, particularly in patients with SAD, which provides supportive evidence for the need to pay attention to small airway function during the management of pediatric asthmatic patients.

Chronic airway inflammation, which affecting both the large and small airways, is a major factor in the development of asthma and is responsible for causing airflow limitation [1]. Guidelines [2, 25] recommend that FEV1 and FEV1/FVC serve as indices in estimating airway obstruction. However, the airflow limitation observed in children differs from that in adults. Several studies demonstrated that as the disease progresses, children were less likely to experience fixed airflow reduction because of the relatively short course of the disease and their FEV_1_% values were not easily impaired [15]. Our results showed that 91.5% (779 of 851) patients had FEV_1_% ≥ 80%, but had varying degrees of reduced terminal airflow, with the highest abnormal rate observed for FEF75%, which was consistent with previous studies [34, 35]. This phenomenon suggests that the small airway dysfunction is involved in the early stages of asthma, even in the absence of obvious large airway impairment. Moreover, small airway function parameters appear to be more sensitive in estimating airway dysfunction in asthmatic children. Of these preschool asthmatic children, 19.5% (166 of 851) had small airway dysfunction, which is much lower than that in the adult studies (50 -90%) [18]. This difference is likely related to different physiological parameters used to assess small airway function. Additionally, small airways are major sites of persistent type 2 inflammation and airway remodeling, which relates to more loss of lung function with aging [14, 35]. Our study also found lung function impairment was more significant in elder asthmatic children. In addition, compared with children with NLF, children with SAD had significantly decreased FEV_1_%, FEV_1_/FVC%, and PEF%. Further analysis demonstrated that FEV_1_%, FEF50%, FEF75% and FEF25-75% were near the lower limit of normal values in patients with LAD with or without a single abnormal small airway parameter. As FEV_1_ reflects both flow and volume components, airflow limitation may induce a decrease in FEF75%, FEF50% or FEF25-75%. This phenomenon was more pronounced in the LSAD groups, in which small airway function decreased remarkably. To some extent, this result suggests small airways involvement is presented in varying degrees in asthmatic patients, particularly in those with severe asthmatic patients.

Our results indicated that there were similar positive correlations between small and large airway function parameters study, which were consistent with other studies [16, 21, 34]. In addition, the variables are not likely to form a complete straight-line trend from the correlation curve fitting diagrams. The curve is steeper in the stage of small airway function decline, and as the small airway function rises to normal, the curve tends to be gentler. These findings suggest that inflammation affects both large and small airway airflow reduction to different degrees and the process of airway obstruction may be distributed unevenly. Pathological studies in asthmatics have shown airway inflammation is a heterogeneous process. Small airways are the major sites where more inflammatory cells (such as T lymphocytes, macrophages, and eosinophils) infiltrated than that in large airways [36–38]. The severe airflow limitation is in line with persistent airway inflammation. Those inflammatory cells accumulate in small airways and involve large airways and alveoli gradually, which could damage airway elastic tissue via the secretion of perforins and granzyme, contributing to airway dysfunction and remodeling [39–41]. Additionally, small airway dysfunction could lead to alterations in the pressure and flow within the airways, which increases shear stresses on the bronchial epithelium and promotes airway remodeling [42, 43]. Small airway dysfunction could also lead to changes in lung mechanics, such as reduced lung compliance, which can cause increased stress on the larger airways [44, 45]. These interrelated mechanisms may explain why small airway dysfunction contributes to the development of large airway dysfunction. However, the pathophysiological relationship between small airway dysfunction and large airway impairment is complex and multifactorial. Future researches are required to elucidate the precise mechanisms involved. As small airways remodeling begins at early stage, anti-asthma treatment, particularly for those with small airway function impairment, may contribute to improved large airway function in the early stage and better-preserved lung function into adulthood. Randomized controlled trials have shown approximately half of extrafine-formulation, one of the small particle sizes of inhaled drugs that can deposit in the peripheral airway, can achieve similar functional outcomes compared with non-extrafine formulations [46, 47]. However, there is currently little direct strong evidence in this area in asthmatic children.

Evidence have shown the decline of FEV_1_ is an independent risk factor for asthma exacerbation, and GINA has provided detailed recommendations for FEV_1_ and PEF as indices for assessing disease conditions and treatment [2]. However, these parameters do not reflect small airway function well [17], especially in asthmatic children [16]. FEF25-75% and FEF50% seem to be better parameters for reflecting small airway function, airflow obstruction and disease severity in asthmatic children than FEV_1_% and FEV_1_/FVC% [15, 34, 48]. Our findings also showed small airway parameters (FEF25-75%, FEF50%, FEF75%) were more strongly correlated with airflow limitation and AHR in preschool asthmatic children. Nevertheless, a large retrospective study [22] showed that FEF25-75% and FEF75% fail to contribute useful information to the clinical assessment of disease severity. This large study cohort was characterized by greater disease heterogeneity, and 71% of the 3 to 10-year-old children had artifacts in the flow-volume curve or did not achieve an individual optimal inspiratory or expiratory status, which could affect the lung function results and research conclusion. Besides, the study did not further investigate the relationship between small airway function and the intensity of AHR, which may be the reason for the inconsistency with our findings.

Airway hyperresponsiveness is a characteristic feature of the asthma development that has been demonstrated to be associated with airway inflammation and small airway ventilation heterogeneity [49]. Research indicates small airway dysfunction, independently of FEV_1_, is related to the severity of AHR in asthma [50]. FEF25-75% was found to be highly related to methacholine responsiveness and the slope of the methacholine dose-response in asthmatic children with normal FEV_1_% [51, 52]. Moreover, FEF50% is an independent predictor of the provocative dose of histamine-associate 20% fall in FEV_1_ [50]. Our findings are consistent with previous studies [50–52] that FEF25-75% and FEF50% have higher correlation coefficients with PC_20_ in asthmatic children compared with FEV_1_%. And a higher level of AHR and airflow obstruction were observed in children with SAD compared to those in NLF group, even though their FEV_1_% was comparable. Thus, small airway function parameters may more accurately reflect the airway function status. Furthermore, our results showed that children had the worst clinical status, including a highest degree of airflow obstruction and AHR when the small airway function was significantly impaired with the presence of FEV_1_% decreased (LSAD group). These findings suggest severe SAD may influence large airway dysfunction and thus contributes to worse clinical manifestation.

To our knowledge, this study complied with our published protocol [53] was the first to investigate the role of small airway dysfunction in a large group of preschool asthmatic children using spirometry. In addition, instead of using one small airway parameter, we combined all three small airway functional parameters of spirometry to evaluate small airway dysfunction and studied the complex correlation of spirometry parameters in preschool asthmatic children. However, our research was a cross-sectional study which can only reflect the current state of lung function in preschool children and cannot explain the effect of persistent SAD on the development of large airway function and the prognosis of the disease. Moreover, as data on confounding factors such as management, passive smoking, exposure to PM2.5 and physical inactivity were lacking [54], we cannot exclude the potential effects of these confounding factors on spirometry. Future studies should consider these information to better interpret the impacts on spirometry parameters.

## Conclusion

Overall, our findings showed small airway dysfunction is strongly associated with abnormal large airway function, airflow obstruction and AHR in preschool asthmatic children. FEF25-75%, FEF50% and FEF75% are sensitive parameters in reflecting airway dysfunction and AHR in asthmatic preschool patients with normal FEV_1_%. These small airway function parameters could be used as supplementary indicators in the assessment of children with large airway dysfunction. Therefore, management strategies for preschool asthmatic children, especially those with normal FEV_1_%, should focus on small airway function.

## Electronic supplementary material

Below is the link to the electronic supplementary material.

Supplementary Material 1

Supplementary Material 2

## Data Availability

Data are available on request to the corresponding author.

## Abbreviations

AHR: Airway hyperresponsiveness
ATS: American Thoracic Society
AUC: Area under the curve
BMI: Body mass index
ERS: European Respiratory Society
FEV_1_: Forced expiratory volume in 1 s in predicted
FEV_1_/FVC: Forced expiratory volume in 1 s/forced expiratory vital capacity ratio
FEF50: Forced expiratory flow at 50% of forced vital capacity predicted
FEF75: Forced expiratory flow at 75% of forced vital capacity predicted
FEF25-75: Forced expiratory flow between 25% and 75% of forced vital capacity predicted
GINA: Global Initiative for Asthma
IQR: Interquartile range
LAD: Large airway dysfunction
LSAD: Large and small airway dysfunction
NLF: Normal lung function
PEF: Peak expiratory flow
ROC: Receiver operating characteristic
SAD: Small airway dysfunction
PC_20_: Value of the provocative concentration of methacholine that caused a 20% fall in FEV_1_
