# Supplementary figures and images for: The role of small airway function parameters in preschool asthmatic children
Source: BMC Pulm Med. 2023 Jun 20;23:219. doi: 10.1186/s12890-023-02515-3 (PMC10283187; doi:10.1186/s12890-023-02515-3)

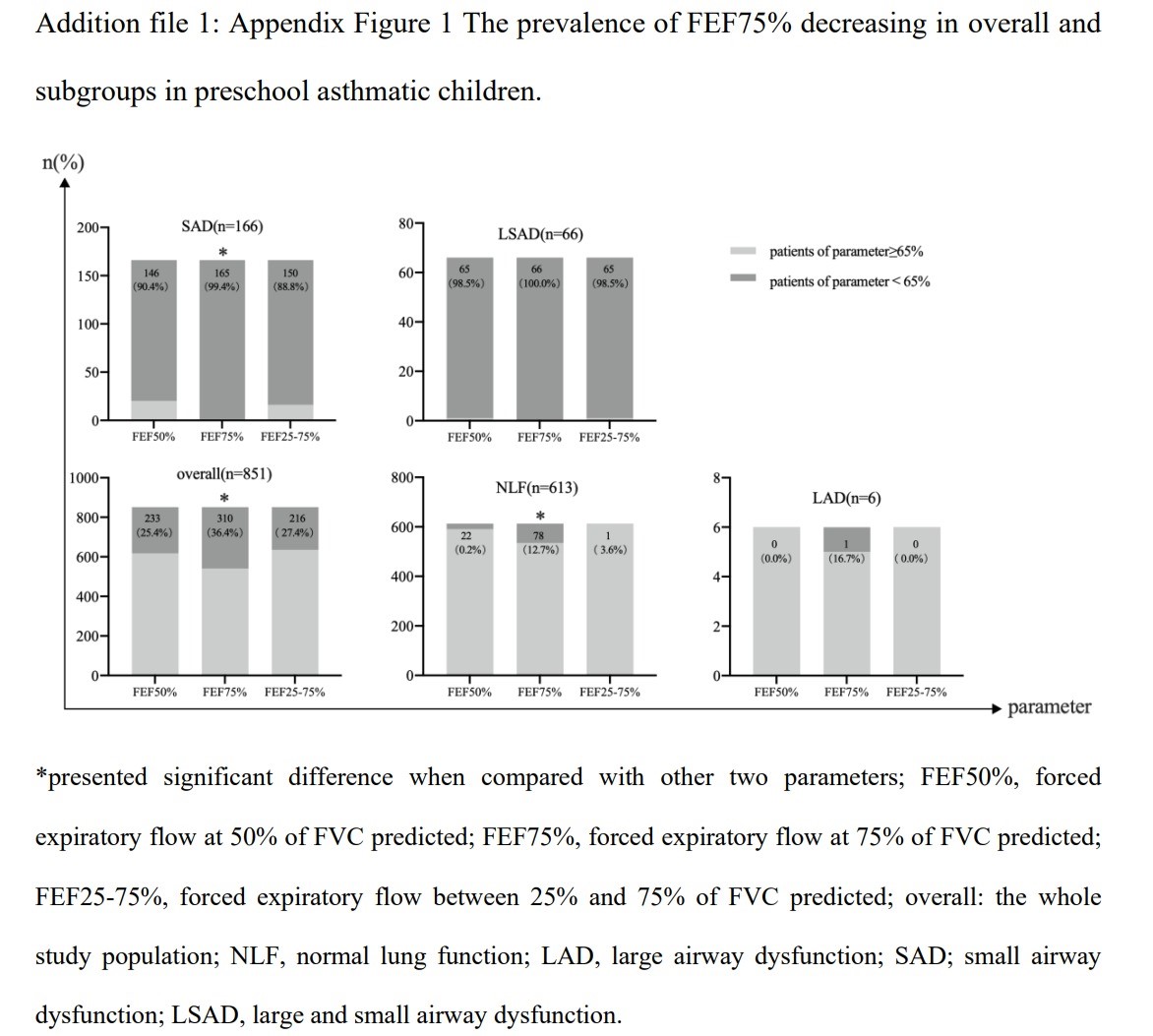

Supplement: Supplementary file 1 — Supplementary Material 1 [file 12890_2023_2515_MOESM1_ESM.jpg]
