# Supplementary material for: The role of small airway function parameters in preschool asthmatic children
Source: BMC Pulm Med. 2023 Jun 20;23:219. doi: 10.1186/s12890-023-02515-3 (PMC10283187; doi:10.1186/s12890-023-02515-3)
Supplement: Supplementary file 2 — Supplementary Material 2 [file 12890_2023_2515_MOESM2_ESM.docx]

| Addition file 2: Appendix table 1 The correlation of FEV_1_% with FEF25-75%, FEF50% and FEF75% in groups and comparison of correlation coefficients. | | | | | | | | | | |
| --- | --- | --- | --- | --- | --- | --- | --- | --- | --- | --- |
|  | Overall (n=851)  r (95%CI) | *p* | NLF (n=613)  r (95%CI) | *p* | LAD(n=6)  r (95%CI) | *p* | SAD (n=166)  r (95%CI) | *p* | LSAD (n=66)  r (95%CI) | *p* |
| FEF25-75% | 0.670 (0.629-0.710) | ＜0.001 | 0.419 (0.344-0.485) | ＜0.001 | 0.429 (-0.800-1.000) | 0.397 | 0.469 (0.338-0.573) | ＜0.001 | 0.553 (0.364-0.693) | ＜0.001 |
| FEF50% | 0.658 (0.612-0.698) | ＜0.001 | 0.399 (0.328-0.468) | ＜0.001 | 0.371 (-0.742-1.000) | 0.468 | 0.363 (0.216-0.495) | ＜0.001 | 0.491 (0.287-0.666) | ＜0.001 |
| FEF75% | 0.609 (0.562-0.655) | ＜0.001 | 0.315 (0.234-0.390) | ＜0.001 | 0.314 (-1.000-1.000) | 0.544 | 0.375 (0.233-0.499) | ＜0.001 | 0.479 (0.284-0.627) | ＜0.001 |
| FEV_1_%, forced expiratory volume in 1 second in predicted; FEF25-75%, forced expiratory flow between 25% and 75% of FVC predicted; FEF50%, forced expiratory flow at 50% of FVC predicted; FEF75%, forced expiratory flow at 75% of FVC predicted; Overall, the whole study population; NLF, normal lung function; LAD, large airway dysfunction; SAD; small airway dysfunction; LSAD, large and small airway dysfunction | | | | | | | | | | |

| Addition file 3: Appendix table 2 The correlation of FEV_1_/FVC% with FEF25-75%, FEF50% and FEF75% in groups. | | | | | | | | | | |
| --- | --- | --- | --- | --- | --- | --- | --- | --- | --- | --- |
|  | Overall (n=851)  r (95%CI) | *p* | NLF (n=613)  r (95%CI) | *p* | LAD(n=6)  r (95%CI) | *p* | SAD (n=166)  r (95%CI) | *p* | LSAD (n=66)  r (95%CI) | *p* |
| FEF25-75% | 0.812 (0.786-0.838) | ＜0.001 | 0.685 (0.640-0.725) | ＜0.001 | 0.886 (0.200-1.000) | 0.019 | 0.630 (0.526-0.712) | ＜0.001 | 0.571 (0.352-0.756) | 0.004 |
| FEF50% | 0.751 (0.720-0.783) | ＜0.001 | 0.562 (0.507-0.615) | ＜0.001 | 0.886 (0.200-1.000) | 0.019 | 0.572 (0.451-0.675) | ＜0.001 | 0.547 (0.308-0.746) | ＜0.001 |
| FEF75% | 0.871 (0.851-0.888) | ＜0.001 | 0.811 (0.780-0.838) | ＜0.001 | 0.943 (0.500-1.000) | 0.005 | 0.601 (0.495-0.701) | ＜0.001 | 0.593 (0.365-0.772) | ＜0.001 |
| FEV_1_/ FVC%, FEV1/ FVC%, forced expiratory vital capacity/forced expiratory vital capacity ratio; FEF25-75%, forced expiratory flow between 25% and 75% of FVC predicted; FEF50%, forced expiratory flow at 50% of FVC predicted; FEF75%, forced expiratory flow at 75% of FVC predicted; Overall, the whole study population; NLF, normal lung function; LAD, large airway dysfunction; SAD; small airway dysfunction; LSAD, large and small airway dysfunction | | | | | | | | | | |

| Addition file 4: Appendix table 3 The correlation of PEF% with FEF25-75%, FEF50% and FEF75% in groups. | | | | | | | | | | |
| --- | --- | --- | --- | --- | --- | --- | --- | --- | --- | --- |
|  | Overall (n=851)  r (95%CI) | *p* | NLF (n=613)  r (95%CI) | *p* | LAD(n=6)  r (95%CI) | *p* | SAD (n=166)  r (95%CI) | *p* | LSAD (n=66)  r (95%CI) | *p* |
| FEF25-75% | 0.626 (0.581-0.669) | ＜0.001 | 0.484 (0.418-0.547) | ＜0.001 | 0.714 (-0.091-1.000) | 0.111 | 0.218 (0.061-0.372) | 0.005 | 0.629 (0.458-0.760) | ＜0.001 |
| FEF50% | 0.635 (0.598-0.676) | ＜0.001 | 0.502 (0.441-0.562) | ＜0.001 | 0.886 (0.200-1.000) | 0.019 | 0.192 (0.049-0.348) | 0.013 | 0.544 (0.353-0.713) | ＜0.001 |
| FEF75% | 0.530 (0.481-0.579) | ＜0.001 | 0.314 (0.232-0.385) | ＜0.001 | 0.771 (0.000-1.000) | 0.072 | 0.075 (-0.086-0.237) | 0.338 | 0.470 (0.270-0.638) | ＜0.001 |
| PEF%, peak expiratory flow in predicted; FEF25-75%, forced expiratory flow between 25% and 75% of FVC predicted; FEF50%, forced expiratory flow at 50% of FVC predicted; FEF75%, forced expiratory flow at 75% of FVC predicted; Overall, the whole study population; NLF, normal lung function; LAD, large airway dysfunction; SAD; small airway dysfunction; LSAD, large and small airway dysfunction | | | | | | | | | | |

| Addition file 5: Appendix table 4 (A to I) Curve fitting models for the relationships between spirometric parameters. | | | | | | | | | | | | | | | | | | | | | | | | |
| --- | --- | --- | --- | --- | --- | --- | --- | --- | --- | --- | --- | --- | --- | --- | --- | --- | --- | --- | --- | --- | --- | --- | --- | --- |
| (A) Curve fitting models for the relationship between FEV_1_% and FEF25-75% | | | | | | | | | | | | | | | | | | | | | | | | |
|  | | Overall（N=851） | | | | NLF（N=613） | | | | | LAD（N=6） | | | | SAD（N=166） | | | | | LSAD（N=66） | | | | |
| Model | | Adj. R^2^ | | *p* | | Adj. R^2^ | | *p* | | | Adj. R^2^ | | *p* | | Adj. R^2^ | | *p* | | | Adj. R^2^ | | *p* | | |
| Linear | | 0.490 | | ＜0.001 | | 0.216 | | ＜0.001 | | | 0.553 | | 0.055 | | 0.213 | | ＜0.001 | | | 0.324 | | ＜0.001 | | |
| Logarithmic | | 0.545 | | ＜0.001 | | 0.214 | | ＜0.001 | | | 0.586 | | 0.047 | | 0.213 | | ＜0.001 | | | 0.367 | | ＜0.001 | | |
| Inverse | | 0.536 | | ＜0.001 | | 0.208 | | ＜0.001 | | | 0.616 | | 0.040 | | 0.212 | | ＜0.001 | | | 0.388 | | ＜0.001 | | |
| Quadratic | | 0.535 | | ＜0.001 | | 0.214 | | ＜0.001 | | | 0.725 | | 0.067 | | 0.209 | | ＜0.001 | | | 0.380 | | ＜0.001 | | |
| Cubic | | 0.557 | | ＜0.001 | | 0.214 | | ＜0.001 | | | 0.835 | | 0.067 | | 0.209 | | ＜0.001 | | | 0.372 | | ＜0.001 | | |
| Compound | | 0.472 | | ＜0.001 | | 0.208 | | ＜0.001 | | | 0.564 | | 0.052 | | 0.215 | | ＜0.001 | | | 0.307 | | ＜0.001 | | |
| Power | | 0.561 | | ＜0.001 | | 0.209 | | ＜0.001 | | | 0.597 | | 0.044 | | 0.216 | | ＜0.001 | | | 0.360 | | ＜0.001 | | |
| S | | 0.599 | | ＜0.001 | | 0.205 | | ＜0.001 | | | 0.628 | | 0.037 | | 0.215 | | ＜0.001 | | | 0.396 | | ＜0.001 | | |
| Growth | | 0.472 | | ＜0.001 | | 0.208 | | ＜0.001 | | | 0.564 | | 0.052 | | 0.215 | | ＜0.001 | | | 0.307 | | ＜0.001 | | |
| Exponential | | 0.472 | | ＜0.001 | | 0.208 | | ＜0.001 | | | 0.651 | | 0.052 | | 0.215 | | ＜0.001 | | | 0.307 | | ＜0.001 | | |
| Logistic | | 0.472 | | ＜0.001 | | 0.208 | | ＜0.001 | | | 0.564 | | 0.052 | | 0.215 | | ＜0.001 | | | 0.307 | | ＜0.001 | | |
| FEV_1_%, forced expiratory volume in 1 second in predicted; FEF25-75%, forced expiratory flow between 25% and 75% of FVC predicted; Overall, the whole study population; NLF, normal lung function; LAD, large airway dysfunction; SAD; small airway dysfunction; LSAD, large and small airway dysfunction; adj. R^2^, adjusted R square. | | | | | | | | | | | | | | | | | | | | | | | | |
| (B) Curve fitting models for the relationship between FEV_1_/FVC% and FEF25-75% | | | | | | | | | | | | | | | | | | | | | | | | |
|  | Overall（N=851） | | | | NLF（N=613） | | | | | LAD（N=6） | | | | | | SAD（N=166） | | | | | LSAD（N=66） | | | |
| Model | Adj. R^2^ | | | *p* | Adj. R^2^ | | | *p* | | Adj. R^2^ | | | | *p* | | Adj. R^2^ | | *p* | | | Adj. R^2^ | *p* | | |
| Linear | 0.621 | | | ＜0.001 | 0.421 | | | ＜0.001 | | 0.516 | | | | 0.066 | | 0.393 | | ＜0.001 | | | 0.315 | ＜0.001 | | |
| Logarithmic | 0.656 | | | ＜0.001 | 0.444 | | | ＜0.001 | | 0.507 | | | | 0.068 | | 0.408 | | ＜0.001 | | | 0.300 | ＜0.001 | | |
| Inverse | 0.583 | | | ＜0.001 | 0.457 | | | ＜0.001 | | 0.496 | | | | 0.072 | | 0.417 | | ＜0.001 | | | 0.263 | ＜0.001 | | |
| Quadratic | 0.669 | | | ＜0.001 | 0.461 | | | ＜0.001 | | 0.373 | | | | 0.231 | | 0.423 | | ＜0.001 | | | 0.304 | ＜0.001 | | |
| Cubic | 0.671 | | | ＜0.001 | 0.461 | | | ＜0.001 | | 0.373 | | | | 0.231 | | 0.424 | | ＜0.001 | | | 0.294 | ＜0.001 | | |
| Compound | 0.609 | | | ＜0.001 | 0.415 | | | ＜0.001 | | 0.524 | | | | 0.063 | | 0.400 | | ＜0.001 | | | 0.323 | ＜0.001 | | |
| Power | 0.655 | | | ＜0.001 | 0.440 | | | ＜0.001 | | 0.516 | | | | 0.066 | | 0.416 | | ＜0.001 | | | 0.313 | ＜0.001 | | |
| S | 0.597 | | | ＜0.001 | 0.454 | | | ＜0.001 | | 0.505 | | | | 0.069 | | 0.426 | | ＜0.001 | | | 0.281 | ＜0.001 | | |
| Growth | 0.609 | | | ＜0.001 | 0.415 | | | ＜0.001 | | 0.524 | | | | 0.063 | | 0.400 | | ＜0.001 | | | 0.323 | ＜0.001 | | |
| Exponential | 0.609 | | | ＜0.001 | 0.415 | | | ＜0.001 | | 0.524 | | | | 0.063 | | 0.400 | | ＜0.001 | | | 0.323 | ＜0.001 | | |
| Logistic | 0.609 | | | ＜0.001 | 0.415 | | | ＜0.001 | | 0.524 | | | | 0.063 | | 0.400 | | ＜0.001 | | | 0.323 | ＜0.001 | | |
| FEV_1_%, forced expiratory volume in 1 second in predicted; FEV_1_/ FVC%, FEV_1_/forced expiratory vital capacity in predicted; FEF25-75%, forced expiratory flow between 25% and 75% of FVC predicted; Overall, the whole study population; NLF, normal lung function; LAD, large airway dysfunction; SAD; small airway dysfunction; LSAD, large and small airway dysfunction; adj. R^2^, adjusted R square. | | | | | | | | | | | | | | | | | | | | | | | | |
| (C) Curve fitting models for the relationship between PEF% and FEF25-75%. | | | | | | | | | | | | | | | | | | | | | | | | |
|  | Overall（N=851） | | | | NLF（N=613） | | | | LAD（N=6） | | | | | | | SAD（N=166） | | | | | LSAD（N=66） | | | |
| Model | Adj. R^2^ | | *p* | | Adj. R^2^ | | *p* | | Adj. R^2^ | | | *p* | | | | Adj. R^2^ | | *p* | | | Adj. R^2^ | *p* | | |
| Linear | 0.449 | | ＜0.001 | | 0.284 | | ＜0.001 | | 0.161 | | | 0.234 | | | | 0.066 | | ＜0.001 | | | 0.390 | ＜0.001 | | |
| Logarithmic | 0.456 | | ＜0.001 | | 0.273 | | ＜0.001 | | 0.172 | | | 0.226 | | | | 0.064 | | 0.001 | | | 0.376 | ＜0.001 | | |
| Inverse | 0.412 | | ＜0.001 | | 0.257 | | ＜0.001 | | 0.182 | | | 0.219 | | | | 0.061 | | 0.001 | | | 0.340 | ＜0.001 | | |
| Quadratic | 0.452 | | ＜0.001 | | 0.288 | | ＜0.001 | | -0.051 | | | 0.065 | | | | 0.002 | | ＜0.001 | | | 0.380 | ＜0.001 | | |
| Cubic | 0.471 | | ＜0.001 | | 0.288 | | ＜0.001 | | -0.051 | | | 0.500 | | | | 0.066 | | ＜0.001 | | | 0.371 | ＜0.001 | | |
| Compound | 0.450 | | ＜0.001 | | 0.277 | | ＜0.001 | | 0.162 | | | 0.234 | | | | 0.065 | | 0.001 | | | 0.382 | ＜0.001 | | |
| Power | 0.488 | | ＜0.001 | | 0.270 | | ＜0.001 | | 0.173 | | | 0.226 | | | | 0.063 | | 0.001 | | | 0.381 | ＜0.001 | | |
| S | 0.478 | | ＜0.001 | | 0.257 | | ＜0.001 | | 0.184 | | | 0.218 | | | | 0.061 | | 0.001 | | | 0.358 | ＜0.001 | | |
| Growth | 0.450 | | ＜0.001 | | 0.277 | | ＜0.001 | | 0.162 | | | 0.234 | | | | 0.065 | | 0.001 | | | 0.382 | ＜0.001 | | |
| Exponential | 0.450 | | ＜0.001 | | 0.277 | | ＜0.001 | | 0.162 | | | 0.234 | | | | 0.065 | | 0.001 | | | 0.382 | ＜0.001 | | |
| Logistic | 0.450 | | ＜0.001 | | 0.277 | | ＜0.001 | | 0.162 | | | 0.234 | | | | 0.065 | | 0.001 | | | 0.382 | ＜0.001 | | |
| PEF%, peak expiratory flow in predicted; FEF25-75%, forced expiratory flow between 25% and 75% of FVC predicted; Overall, the whole study population; NLF, normal lung function; LAD, large airway dysfunction; SAD; small airway dysfunction; LSAD, large and small airway dysfunction; adj. R^2^, adjusted R square. | | | | | | | | | | | | | | | | | | | | | | | | |
| (D) Curve fitting models for the relationship between FEV_1_% and FEF50% | | | | | | | | | | | | | | | | | | | | | | | | |
|  | | Overall（N=851） | | | | NLF（N=613） | | | | | LAD（N=6） | | | | | SAD（N=166） | | | | | LSAD（N=66） | | | |
| Model | | Adj. R^2^ | | *p* | | Adj. R^2^ | | *p* | | | Adj. R^2^ | | | *p* | | Adj. R^2^ | | | *p* | | Adj. R^2^ | | *p* | |
| Linear | | 0.470 | | ＜0.001 | | 0.194 | | ＜0.001 | | | -0.039 | | | 0.419 | | 0.103 | | | ＜0.001 | | 0.263 | | ＜0.001 | |
| Logarithmic | | 0.521 | | ＜0.001 | | 0.189 | | ＜0.001 | | | -0.032 | | | 0.410 | | 0.109 | | | ＜0.001 | | 0.287 | | ＜0.001 | |
| Inverse | | 0.519 | | ＜0.001 | | 0.179 | | ＜0.001 | | | -0.024 | | | 0.401 | | 0.111 | | | ＜0.001 | | 0.295 | | ＜0.001 | |
| Quadratic | | 0.515 | | ＜0.001 | | 0.193 | | ＜0.001 | | | -0.204 | | | 0.614 | | 0.114 | | | ＜0.001 | | 0.279 | | ＜0.001 | |
| Cubic | | 0.531 | | ＜0.001 | | 0.191 | | ＜0.001 | | | -0.204 | | | 0.614 | | 0.116 | | | ＜0.001 | | 0.271 | | ＜0.001 | |
| Compound | | 0.454 | | ＜0.001 | | 0.188 | | ＜0.001 | | | -0.036 | | | 0.415 | | 0.106 | | | ＜0.001 | | 0.254 | | ＜0.001 | |
| Power | | 0.534 | | ＜0.001 | | 0.185 | | ＜0.001 | | | -0.029 | | | 0.406 | | 0.111 | | | ＜0.001 | | 0.287 | | ＜0.001 | |
| S | | 0.572 | | ＜0.001 | | 0.177 | | ＜0.001 | | | -0.021 | | | 0.397 | | 0.114 | | | ＜0.001 | | 0.307 | | ＜0.001 | |
| Growth | | 0.454 | | ＜0.001 | | 0.188 | | ＜0.001 | | | -0.036 | | | 0.415 | | 0.106 | | | ＜0.001 | | 0.254 | | ＜0.001 | |
| Exponential | | 0.454 | | ＜0.001 | | 0.188 | | ＜0.001 | | | -0.036 | | | 0.415 | | 0.106 | | | ＜0.001 | | 0.254 | | ＜0.001 | |
| Logistic | | 0.454 | | ＜0.001 | | 0.188 | | ＜0.001 | | | -0.036 | | | 0.415 | | 0.106 | | | ＜0.001 | | 0.254 | | ＜0.001 | |
| FEV_1_%, forced expiratory volume in 1 second in predicted; FEF50%, forced expiratory flow at 50% of FVC predicted; Overall, the whole study population; NLF, normal lung function; LAD, large airway dysfunction; SAD; small airway dysfunction; LSAD, large and small airway dysfunction; adj. R^2^, adjusted R square. | | | | | | | | | | | | | | | | | | | | | | | | |
| (E) Curve fitting models for the relationship between FEV_1_/FVC% and FEF50% | | | | | | | | | | | | | | | | | | | | | | | |  |
|  | Overall（N=851） | | | | NLF（N=613） | | | | LAD（N=6） | | | | | | | SAD（N=166） | | | | | LSAD（N=66） | | |  |
| Model | Adj. R^2^ | | *p* | | Adj. R^2^ | | *p* | | Adj. R^2^ | | | *p* | | | | Adj. R^2^ | | *p* | | | Adj. R^2^ | *p* | |  |
| Linear | 0.550 | | ＜0.001 | | 0.297 | | ＜0.001 | | 0.850 | | | 0.006 | | | | 0.350 | | ＜0.001 | | | 0.307 | ＜0.001 | |  |
| Logarithmic | 0.593 | | ＜0.001 | | 0.307 | | ＜0.001 | | 0.864 | | | 0.005 | | | | 0.359 | | ＜0.001 | | | 0.309 | ＜0.001 | |  |
| Inverse | 0.556 | | ＜0.001 | | 0.306 | | ＜0.001 | | 0.876 | | | 0.004 | | | | 0.364 | | ＜0.001 | | | 0.287 | ＜0.001 | |  |
| Quadratic | 0.603 | | ＜0.001 | | 0.309 | | ＜0.001 | | 0.937 | | | 0.007 | | | | 0.357 | | ＜0.001 | | | 0.301 | ＜0.001 | |  |
| Cubic | 0.602 | | ＜0.001 | | 0.308 | | ＜0.001 | | 0.938 | | | 0.007 | | | | 0.357 | | ＜0.001 | | | 0.292 | ＜0.001 | |  |
| Compound | 0.543 | | ＜0.001 | | 0.294 | | ＜0.001 | | 0.846 | | | 0.006 | | | | 0.360 | | ＜0.001 | | | 0.328 | ＜0.001 | |  |
| Power | 0.597 | | ＜0.001 | | 0.305 | | ＜0.001 | | 0.860 | | | 0.005 | | | | 0.371 | | ＜0.001 | | | 0.336 | ＜0.001 | |  |
| S | 0.573 | | ＜0.001 | | 0.305 | | ＜0.001 | | 0.873 | | | 0.004 | | | | 0.377 | | ＜0.001 | | | 0.319 | ＜0.001 | |  |
| Growth | 0.543 | | ＜0.001 | | 0.294 | | ＜0.001 | | 0.846 | | | 0.006 | | | | 0.360 | | ＜0.001 | | | 0.328 | ＜0.001 | |  |
| Exponential | 0.543 | | ＜0.001 | | 0.294 | | ＜0.001 | | 0.846 | | | 0.006 | | | | 0.360 | | ＜0.001 | | | 0.328 | ＜0.001 | |  |
| Logistic | 0.543 | | ＜0.001 | | 0.294 | | ＜0.001 | | 0.846 | | | 0.006 | | | | 0.360 | | ＜0.001 | | | 0.328 | ＜0.001 | |  |
| FEV_1_%, forced expiratory volume in 1 second in predicted; FEV_1_/ FVC%, FEV_1_/forced expiratory vital capacity in predicted; FEF50%, forced expiratory flow at 50% of FVC predicted; Overall, the whole study population; NLF, normal lung function; LAD, large airway dysfunction; SAD; small airway dysfunction; LSAD, large and small airway dysfunction; adj. R^2^, adjusted R square. | | | | | | | | | | | | | | | | | | | | | | | |  |
| (F) Curve fitting models for the relationship between PEF% and FEF50% | | | | | | | | | | | | | | | | | | | | | | | |  |
|  | Overall（N=851） | | | | NLF（N=613） | | | | LAD（N=6） | | | | | | | SAD（N=166） | | | | | LSAD（N=66） | | |  |
| Model | Adj. R^2^ | | *p* | | Adj. R^2^ | | *p* | | Adj. R^2^ | | | *p* | | | | Adj. R^2^ | | *p* | | | Adj. R^2^ | *p* | |  |
| Linear | 0.461 | | ＜0.001 | | 0.307 | | ＜0.001 | | 0.446 | | | 0.088 | | | | 0.038 | | 0.007 | | | 0.322 | ＜0.001 | |  |
| Logarithmic | 0.463 | | ＜0.001 | | 0.289 | | ＜0.001 | | 0.463 | | | 0.083 | | | | 0.042 | | 0.005 | | | 0.302 | ＜0.001 | |  |
| Inverse | 0.419 | | ＜0.001 | | 0.265 | | ＜0.001 | | 0.479 | | | 0.077 | | | | 0.044 | | 0.004 | | | 0.265 | ＜0.001 | |  |
| Quadratic | 0.462 | | ＜0.001 | | 0.317 | | ＜0.001 | | 0.559 | | | 0.136 | | | | 0.045 | | 0.009 | | | 0.316 | ＜0.001 | |  |
| Cubic | 0.478 | | ＜0.001 | | 0.316 | | ＜0.001 | | 0.559 | | | 0.136 | | | | 0.046 | | 0.008 | | | 0.311 | ＜0.001 | |  |
| Compound | 0.458 | | ＜0.001 | | 0.300 | | ＜0.001 | | 0.440 | | | 0.090 | | | | 0.039 | | 0.006 | | | 0.308 | ＜0.001 | |  |
| Power | 0.489 | | ＜0.001 | | 0.286 | | ＜0.001 | | 0.457 | | | 0.084 | | | | 0.043 | | 0.004 | | | 0.299 | ＜0.001 | |  |
| S | 0.476 | | ＜0.001 | | 0.266 | | ＜0.001 | | 0.474 | | | 0.079 | | | | 0.045 | | 0.003 | | | 0.273 | ＜0.001 | |  |
| Growth | 0.458 | | ＜0.001 | | 0.300 | | ＜0.001 | | 0.440 | | | 0.090 | | | | 0.039 | | 0.006 | | | 0.308 | ＜0.001 | |  |
| Exponential | 0.458 | | ＜0.001 | | 0.300 | | ＜0.001 | | 0.440 | | | 0.090 | | | | 0.039 | | 0.006 | | | 0.308 | ＜0.001 | |  |
| Logistic | 0.458 | | ＜0.001 | | 0.300 | | ＜0.001 | | 0.440 | | | 0.090 | | | | 0.039 | | 0.006 | | | 0.308 | ＜0.001 | |  |
| PEF%, peak expiratory flow in predicted; FEF50%, forced expiratory flow at 50% of FVC predicted; Overall, the whole study population; NLF, normal lung function; LAD, large airway dysfunction; SAD; small airway dysfunction; LSAD, large and small airway dysfunction; adj. R^2^, adjusted R square. | | | | | | | | | | | | | | | | | | | | | | | |  |
| (G) Curve fitting models for the relationship between FEV_1_% and FEF75%. | | | | | | | | | | | | | | | | | | | | | | | |  |
|  | Overall（N=851） | | | | NLF（N=613） | | | | LAD（N=6） | | | | | | | SAD（N=166） | | | | | LSAD（N=66） | | |  |
| Model | Adj. R^2^ | | *p* | | Adj. R^2^ | | *p* | | Adj. R^2^ | | | *p* | | | | Adj. R^2^ | | *p* | | | Adj. R^2^ | *p* | |  |
| Linear | 0.361 | | ＜0.001 | | 0.112 | | ＜0.001 | | 0.401 | | | 0.105 | | | | 0.125 | | ＜0.001 | | | 0.180 | ＜0.001 | |  |
| Logarithmic | 0.444 | | ＜0.001 | | 0.119 | | ＜0.001 | | 0.474 | | | 0.079 | | | | 0.135 | | ＜0.001 | | | 0.195 | ＜0.001 | |  |
| Inverse | 0.459 | | ＜0.001 | | 0.120 | | ＜0.001 | | 0.541 | | | 0.058 | | | | 0.136 | | ＜0.001 | | | 0..190 | ＜0.001 | |  |
| Quadratic | 0.448 | | ＜0.001 | | 0.120 | | ＜0.001 | | 0.634 | | | 0.103 | | | | 0.142 | | ＜0.001 | | | 0.193 | ＜0.001 | |  |
| Cubic | 0.462 | | ＜0.001 | | 0.119 | | ＜0.001 | | 0.634 | | | 0.103 | | | | 0.159 | | ＜0.001 | | | 0.183 | 0.001 | |  |
| Compound | 0.346 | | ＜0.001 | | 0.108 | | ＜0.001 | | 0.414 | | | 0.100 | | | | 0.129 | | ＜0.001 | | | 0.162 | ＜0.001 | |  |
| Power | 0.450 | | ＜0.001 | | 0.116 | | ＜0.001 | | 0.488 | | | 0.074 | | | | 0.139 | | ＜0.001 | | | 0.175 | ＜0.001 | |  |
| S | 0.497 | | ＜0.001 | | 0.118 | | ＜0.001 | | 0.554 | | | 0.055 | | | | 0.141 | | ＜0.001 | | | 0.169 | ＜0.001 | |  |
| Growth | 0.346 | | ＜0.001 | | 0.108 | | ＜0.001 | | 0.414 | | | 0.100 | | | | 0.129 | | ＜0.001 | | | 0.162 | ＜0.001 | |  |
| Exponential | 0.346 | | ＜0.001 | | 0.108 | | ＜0.001 | | 0.414 | | | 0.100 | | | | 0.129 | | ＜0.001 | | | 0.162 | ＜0.001 | |  |
| Logistic | 0.346 | | ＜0.001 | | 0.108 | | ＜0.001 | | 0.414 | | | 0.100 | | | | 0.129 | | ＜0.001 | | | 0.162 | ＜0.001 | |  |
| FEV_1_%, forced expiratory volume in 1 second in predicted; FEF75%, forced expiratory flow at 75% of FVC predicted; Overall, the whole study population; NLF, normal lung function; LAD, large airway dysfunction; SAD; small airway dysfunction; LSAD, large and small airway dysfunction; adj. R^2^, adjusted R square. | | | | | | | | | | | | | | | | | | | | | | | |  |
| (H) Curve fitting models for the relationship between FEV1/FVC% and FEF75%. | | | | | | | | | | | | | | | | | | | | | | | |  |
|  | Overall（N=851） | | | | NLF（N=613） | | | | LAD（N=6） | | | | | | | SAD（N=166） | | | | | LSAD（N=66） | | |  |
| Model | Adj. R^2^ | | *p* | | Adj. R^2^ | | *p* | | Adj. R^2^ | | | *p* | | | | Adj. R^2^ | | *p* | | | Adj. R^2^ | *p* | |  |
| Linear | 0.653 | | ＜0.001 | | 0.559 | | ＜0.001 | | 0.641 | | | 0.034 | | | | 0.353 | | ＜0.001 | | | 0.336 | ＜0.001 | |  |
| Logarithmic | 0.721 | | ＜0.001 | | 0.617 | | ＜0.001 | | 0.601 | | | 0.043 | | | | 0.324 | | ＜0.001 | | | 0.353 | ＜0.001 | |  |
| Inverse | 0.652 | | ＜0.001 | | 0.637 | | ＜0.001 | | 0.555 | | | 0.055 | | | | 0.279 | | ＜0.001 | | | 0.349 | ＜0.001 | |  |
| Quadratic | 0.736 | | ＜0.001 | | 0.644 | | ＜0.001 | | 0.597 | | | 0.119 | | | | 0.366 | | ＜0.001 | | | 0.338 | ＜0.001 | |  |
| Cubic | 0.736 | | ＜0.001 | | 0.645 | | ＜0.001 | | 0.596 | | | 0.120 | | | | 0.363 | | ＜0.001 | | | 0.339 | ＜0.001 | |  |
| Compound | 0.630 | | ＜0.001 | | 0.551 | | ＜0.001 | | 0.651 | | | 0.033 | | | | 0.351 | | ＜0.001 | | | 0.330 | ＜0.001 | |  |
| Power | 0.711 | | ＜0.001 | | 0.611 | | ＜0.001 | | 0.611 | | | 0.041 | | | | 0.323 | | ＜0.001 | | | 0.352 | ＜0.001 | |  |
| S | 0.660 | | ＜0.001 | | 0.634 | | ＜0.001 | | 0.566 | | | 0.052 | | | | 0.278 | | ＜0.001 | | | 0.352 | ＜0.001 | |  |
| Growth | 0.630 | | ＜0.001 | | 0.551 | | ＜0.001 | | 0.651 | | | 0.033 | | | | 0.351 | | ＜0.001 | | | 0.330 | ＜0.001 | |  |
| Exponential | 0.630 | | ＜0.001 | | 0.551 | | ＜0.001 | | 0.651 | | | 0.033 | | | | 0.351 | | ＜0.001 | | | 0.330 | ＜0.001 | |  |
| Logistic | 0.630 | | ＜0.001 | | 0.551 | | ＜0.001 | | 0.651 | | | 0.033 | | | | 0.351 | | ＜0.001 | | | 0.330 | ＜0.001 | |  |
| FEV_1_%, forced expiratory volume in 1 second in predicted; FEV_1_/ FVC%, FEV_1_/forced expiratory vital capacity in predicted; FEF75%, forced expiratory flow at 75% of FVC predicted; Overall, the whole study population; NLF, normal lung function; LAD, large airway dysfunction; SAD; small airway dysfunction; LSAD, large and small airway dysfunction; adj. R^2^, adjusted R square. | | | | | | | | | | | | | | | | | | | | | | | |  |
| (I) Curve fitting models for the relationship between PEF% and FEF75% | | | | | | | | | | | | | | | | | | | | | | | |  |
|  | | Overall（N=851） | | | | NLF（N=613） | | | | | LAD（N=6） | | | | | SAD（N=166） | | | | | LSAD（N=66） | | |  |
| Model | | Adj. R^2^ | | *p* | | Adj. R^2^ | | *p* | | | Adj. R^2^ | | | *p* | | Adj. R^2^ | | *p* | | | Adj. R^2^ | *p* | |  |
| Linear | | 0.293 | | ＜0.001 | | 0.114 | | ＜0.001 | | | 0.169 | | | 0.229 | | 0.008 | | 0.129 | | | 0.182 | ＜0.001 | |  |
| Logarithmic | | 0.330 | | ＜0.001 | | 0.112 | | ＜0.001 | | | 0.185 | | | 0.218 | | 0.008 | | 0.124 | | | 0.185 | ＜0.001 | |  |
| Inverse | | 0.318 | | ＜0.001 | | 0.104 | | ＜0.001 | | | 0.200 | | | 0.208 | | 0.008 | | 0.132 | | | 0.168 | ＜0.001 | |  |
| Quadratic | | 0.322 | | ＜0.001 | | 0.113 | | ＜0.001 | | | -0.080 | | | 0.522 | | 0.003 | | 0.299 | | | 0.178 | 0.001 | |  |
| Cubic | | 0.328 | | ＜0.001 | | 0.114 | | ＜0.001 | | | -0.080 | | | 0.522 | | -0.002 | | 0.440 | | | 0.182 | 0.001 | |  |
| Compound | | 0.295 | | ＜0.001 | | 0.111 | | ＜0.001 | | | 0.173 | | | 0.226 | | 0.006 | | 0.160 | | | 0.176 | ＜0.001 | |  |
| Power | | 0.353 | | ＜0.001 | | 0.110 | | ＜0.001 | | | 0.190 | | | 0.214 | | 0.007 | | 0.151 | | | 0.178 | ＜0.001 | |  |
| S | | 0.362 | | ＜0.001 | | 0.102 | | ＜0.001 | | | 0.205 | | | 0.205 | | 0.006 | | 0.156 | | | 0.160 | 0.001 | |  |
| Growth | | 0.295 | | ＜0.001 | | 0.111 | | ＜0.001 | | | 0.173 | | | 0.226 | | 0.006 | | 0.160 | | | 0.176 | ＜0.001 | |  |
| Exponential | | 0.295 | | ＜0.001 | | 0.111 | | ＜0.001 | | | 0.173 | | | 0.226 | | 0.006 | | 0.160 | | | 0.176 | ＜0.001 | |  |
| Logistic | | 0.295 | | ＜0.001 | | 0.111 | | ＜0.001 | | | 0.173 | | | 0.226 | | 0.006 | | 0.160 | | | 0.176 | ＜0.001 | |  |
| PEF%, peak expiratory flow in predicted; FEF75%, forced expiratory flow at 75% of FVC predicted; Overall, the whole study population; NLF, normal lung function; LAD, large airway dysfunction; SAD; small airway dysfunction; LSAD, large and small airway dysfunction; adj. R^2^, adjusted R square. | | | | | | | | | | | | | | | | | | | | | | | |  |
